# Impact of the interval between neoadjuvant concurrent chemoradiotherapy and esophagectomy in the modern era: a population-based propensity-score-matched retrospective cohort study in Asia

**DOI:** 10.1186/s12957-019-1712-7

**Published:** 2019-12-19

**Authors:** Yao-Hung Kuo, Yu-Wen Chien, Pin-Ru Chen, Chun-Lung Feng, Chia-Chin Li, Chun-Ru Chien

**Affiliations:** 1 Department of Radiation Oncology, E-Da Hospital, Kaohsiung, Taiwan; 2 College of Medicine, I-Shou University, Kaohsiung, Taiwan; 3 Department of Public Health, College of Medicine, National Cheng Kung University, Tainan, Taiwan; 4 Department of Chest Surgery, China Medical University Hsinchu Hospital, Hsinchu, Taiwan; 5 Division of Gastroenterology and Hepatology, China Medical University Hsinchu Hospital, Hsinchu, Taiwan; 6 Department of Radiation Oncology, China Medical University Hospital, Taichung, Taiwan; 7 Department of Radiation Oncology, China Medical University Hsinchu Hospital, Hsinchu, Taiwan; 8 Department of Medical Research, China Medical University Hsinchu Hospital, Hsinchu, Taiwan; 9 School of Medicine, College of Medicine, China Medical University, Taichung, Taiwan

**Keywords:** Esophageal squamous cell carcinoma, Neoadjuvant concurrent chemoradiotherapy, Esophagectomy, Interval

## Abstract

**Background:**

Neoadjuvant concurrent chemoradiotherapy (nCCRT) is one of the standard-of-care options for locally advanced esophageal squamous cell carcinoma (LA-ESqCC). The optimal interval between nCCRT and esophagectomy is unknown.

**Methods:**

We constructed a propensity-score-matched [1:1 for long (8–12 weeks) vs short (4–7 weeks) intervals] cohort of LA-ESqCC patients who were diagnosed from 2011 to 2015 and treated with nCCRT via the Taiwan Cancer Registry and related databases. We compared the hazard ratios (HRs) of death using a robust variance estimator. We also evaluated alternative covariables, outcomes, and interval definitions.

**Results:**

Our study population included 80 patients for each group; groups were balanced with respect to the observed covariables. There was no significant difference for the HR of death [1.22; 95% confidence interval 0.78–1.91, *P* = 0.39] when the long interval group was compared to the short interval group. There were also no significant differences when alternative covariables, outcomes, or interval definitions were evaluated.

**Conclusions:**

In this population-based study in modern Asia, we found that for LA-ESqCC patients treated with nCCRT and esophagectomy, overall survival was similar for either long or short intervals between nCCRT and esophagectomy. Randomized controlled trials are needed to verify this finding.

## Background

Esophageal cancer is one of the common causes of cancer mortality worldwide [1]. In contrast to the Western world, where adenocarcinoma is the common histology, squamous cell carcinoma (SqCC) is the predominant histology in Asia [2]. For locally advanced esophageal SqCC (LA-ESqCC), neoadjuvant concurrent chemoradiotherapy (nCCRT) is one of the standard-of-care options [3–6].

However, the optimal interval between nCCRT and esophagectomy is debated in the literature [7]. In clinical practice, some interval is needed for patients to recover from the side effects of nCCRT, but delayed surgery might lead to tumor growth. In the experience of nCCRT for rectal cancer, a randomized controlled trial (RCT) reported that prolongation was associated with a higher pathological complete response (pCR) [8], a well-known good prognostic factor [9]. In contrast, another RCT reported that prolongation led to a similar pCR but a higher morbidity [10].

Regarding nCCRT for esophageal cancer, a systematic review of non-RCTs published in 2018 reported that a long interval (> 7–8 weeks, vs ≤ 7–8 weeks) was associated with higher pCR rates but worse overall survival (OS), both with statistical significance [7]. However, all Asian studies included in this study were based on patients treated almost a decade ago. In addition, the results of individual studies included in this systematic review were variable. Given the abovementioned geographic variation, controversy in this topic, and lack of RCTs, we aimed to compare the OS of LA-ESqCC treated with nCCRT and esophagectomy in modern Asia with either long or short intervals via a population-based propensity-score-matched analysis.

## Methods

### Data source

The Health and Welfare Data Science Center (HWDC), Ministry of Health and Welfare, database is a set of databases providing complete information regarding the Taiwan Cancer Registry (TCR) (data until 2015), the death registry (data until December 31, 2017), and reimbursement data from the National Health Insurance (NHI) (data until December 31, 2016) for the whole Taiwan population, and it is provided by the Bureau of National Health Insurance [11]. The quality of the TCR was reported in 2019 [12]. The NHI research database has also been used in many population-based studies. All of the HWDC data with personal information were deidentified.

### Study population and design

**Fig. 1:**
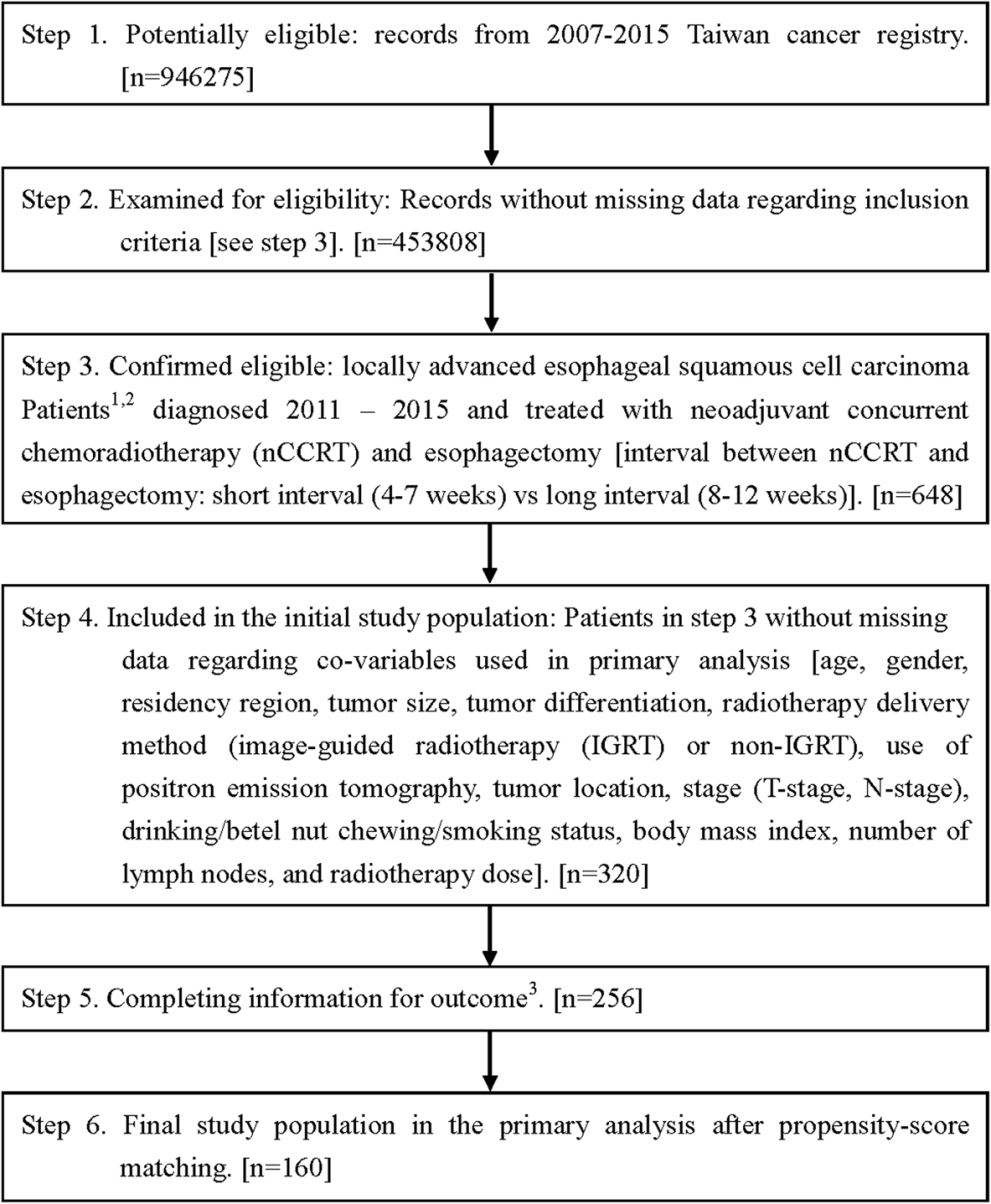
STROBE study flowchart and the number of individuals at each stage of the study. 1: We only included those treated (class 1–2) by any single institution to ensure data consistency. 2: Clinical stage II–III, by the 7th American Joint Committee on Cancer staging. 3: Without missing information in the TCR and death registry

The study flow chart, as suggested in the STROBE statement [13], is depicted in Fig. 1. In this retrospective cohort study, we used the HWDC database to identify LA-ESqCC patients who were diagnosed from 2011 to 2015 and treated with nCCRT (radiotherapy 40–50.4 Gy at a dose per fraction of 1.8–2 Gy) and esophagectomy. nCCRT was defined as concurrent systemic and locoregional therapy with preoperative radiotherapy per the TCR record. Patients with other cancer(s) were excluded. The date of diagnosis was used as the index date. We determined the explanatory variable of interest [interval between nCCRT and esophagectomy (long interval (8–12 weeks) vs short interval (4–7 weeks))] based on the cancer registry data; the primary outcome of interest [OS] and other supplementary outcomes [pCR, 30 and 90 day mortality (since surgery), incidence of local regional recurrence (ILRR), and esophageal cancer mortality (IECM)] were extracted from the TCR or determined via linkage with the death registry. OS was calculated from the date of diagnosis to the date of death or December 31, 2017 (censoring date of the death registry). We also considered other covariables [see the next section] to adjust for potential nonrandomized treatment selection and then constructed a propensity-score (PS)-matched sample (1:1 paired matching) to evaluate the effectiveness of the interval between nCCRT and esophagectomy.

### Other explanatory covariables

We identified patient demographic factors [age, gender, residency region], patient characteristics [drinking, betel nut chewing, smoking, body mass index (BMI)], disease characteristics [tumor size, tumor differentiation, tumor location, clinical T-stage and N-stage], diagnosis method [use of positron emission tomography (PET)], and treatment characteristics [number of lymph nodes removed, radiotherapy (RT) delivery method, RT dose] as potential confounders based on our experiences in clinical practice and modified from our TCR/NHI related study [6]. These covariables were defined as follows. Patient residency was classified as northern Taiwan or elsewhere. The drinking, betel nut chewing, smoking, and use of PET variables were classified as yes or no. The number of lymph nodes was classified as < 15 or ≥ 15. Tumor size was dichotomized by tumors having a diameter ≤ 5 or > 5 cm. Tumor differentiation was classified as well/moderately differentiated or poorly/undifferentiated. Tumor location was classified as cervical or not. Clinical stage was classified as T1–T2 vs T3–T4 for T-stage and negative vs positive for N-stage. RT delivery was classified as image-guided radiotherapy (IGRT) or non-IGRT.

### Statistical analyses

In the primary analysis (PA), we used the propensity score method as advocated in the literature to balance the measured potential confounders [14, 15]. We used a logistic regression model based on all covariables [see the above subsection “Other explanatory covariables”] to evaluate the probability with a long interval [vs a short interval]. Patients were matched on the logit of the propensity score using a caliper of 0.25 standard deviations of the logit of the propensity score via a greedy match algorithm as used in the literature [16]. The standardized difference (SDif) was used to assess the balance of the covariates [17, 18]. We used a robust variance estimator to compare the hazard ratio (HR) of death between PS-matched groups during the entire follow-up period [15] and evaluated the effect of potential unmeasured confounding factor(s) via the *E* value [19]. Binary outcomes (pCR) within the matched pairs were compared using McNemar’s test. We adopted the subdistribution HR via the clustered Fine–Gray model to evaluate ILRR and IECM [20]. Because of the vague [7–8 weeks] cutoff point used in the recent systematic review [7], we used alternative definitions [(1) 4–8 weeks vs 8–12 weeks; (2) 4–7 weeks vs 7–12 weeks] for the interval between nCCRT and esophagectomy to compare the OS as the first and second supplementary analyses (SA-1, SA-2) via separate PS matching. In the third SA (SA-3), we considered additional covariables [including site patient volume [21, 22] plus number of positive lymph node] and outcome [R0 resection], by constructing another PS-matched population for comparison. Although optimal interval was not specified in the recent treatment guideline [3], 4~6 weeks were commonly used in the RCT [23, 24]. Therefore, we performed the fourth SA (SA-4) by constructing additional PS-matched population to only compare 4~6 weeks vs 6~8 weeks. SAS v.9.4 software (SAS Institute, Cary, NC, USA) was used for statistical analyses.

## Results

### Study population

As shown in Fig. 1, we identified 160 eligible PS-matched patients treated with nCCRT and esophagectomy between 2011 and 2015 from 7908 esophageal cancer patients (65% locally advanced) as our primary study population and divided them into two groups [long interval group (*n* = 80) vs short interval group (*n* = 80)]. All covariates were balanced [SDif < 0.25] after matching (Table 1), though some were not balanced before matching.

**Table 1:** Patient characteristics of the study population in the primary analysis

		Unmatched population					Matched study population				
		Short interval (*n* = 169)		Long interval (*n* = 87)			Short interval (*n* = 80)		Long interval (*n* = 80)		
		Number or mean (sd)^†^	(%)^†^	Number or mean (sd)^†^	(%)^†^	SDif^†^	Number or mean (sd)^†^	(%)^†^	Number or mean (sd)^†^	(%)^†^	SDif^†^
Age		54.71 (8.53)		53.21 (8.30)		0.179	53.48 (8.62)		52.94 (8.31)		0.063
Gender	Female	13	(8)	5	(6)	0.078	5	(6)	5	(6)	0
	Male	156	(92)	82	(94)		75	(94)	75	(94)	
Residency	Non-north	110	(65)	46	(53)	0.250	46	(57)	45	(56)	0.025
	North	59	(35)	41	(47)		34	(43)	35	(44)	
Tumor size	≤ 5 cm	61	(36)	43	(49)	0.272	35	(44)	37	(46)	0.050
	> 5 cm	108	(64)	44	(51)		45	(56)	43	(54)	
Tumor differentiation	Poorly/undifferentiated	66	(39)	15	(17)	0.500	15	(19)	15	(19)	0
	Well/moderately	103	(61)	72	(83)		65	(81)	65	(81)	
RT delivery	Non-IGRT	145	(86)	62	(71)	0.360	62	(77)	60	(75)	0.059
	IGRT	24	(14)	25	(29)		18	(23)	20	(25)	
Use of PET	No	14	(8)	7	(8)	0.009	5	(6)	7	(9)	0.095
	Yes	155	(92)	80	(92)		75	(94)	73	(91)	
Tumor location	Cervical	^‡^	^‡^	^‡^	^‡^	0.220	^‡^	^‡^	^‡^	^‡^	0
	Non-cervical	^‡^	^‡^	^‡^	^‡^		^‡^	^‡^	^‡^	^‡^	
T-stage	T1–T2	21	(12)	10	(11)	0.029	10	(13)	10	(13)	0
	T3–T4	148	(88)	77	(89)		70	(87)	70	(87)	
N-stage	Negative	20	(12)	8	(9)	0.086	14	(18)	8	(10)	0.219
	Positive	149	(88)	79	(91)		66	(82)	72	(90)	
Drinking	No	23	(14)	7	(8)	0.180	8	(10)	7	(9)	0.043
	Yes	146	(86)	80	(92)		72	(90)	73	(91)	
Betel nut chewing	No	75	(44)	30	(34)	0.204	29	(36)	28	(35)	0.026
	Yes	94	(56)	57	(66)		51	(64)	52	(65)	
Smoking	No	24	(14)	10	(11)	0.081	10	(13)	10	(13)	0
	Yes	145	(86)	77	(89)		70	(87)	70	(87)	
BMI		22.08 (3.34)		22.69 (4.62)		0.152	21.91 (3.35)		22.39 (3.32)		0.146
Number of lymph nodes	< 15	33	(20)	17	(20)	0	20	(25)	16	(20)	0.120
	≥ 15	136	(80)	70	(80)		60	(75)	64	(80)	
RT dose (Gy)		48.30 (3.30)		46.80 (4.06)		0.405	47.51 (3.59)		47.16 (3.92)		0.094

*BMI* body mass index, *IGRT* image-guided radiotherapy, *nCCRT* neoadjuvant concurrent chemoradiotherapy, *PET* positron emission tomography, *RT* radiotherapy, *sd* standard deviation, *SDif* standardized difference

^†^Rounded

^‡^The exact numbers were not reported because of a Health and Welfare Data Science Center (HWDC) database center policy to avoid numbers in single cells (≤ 2)

### Primary analysis

**Fig. 2:**
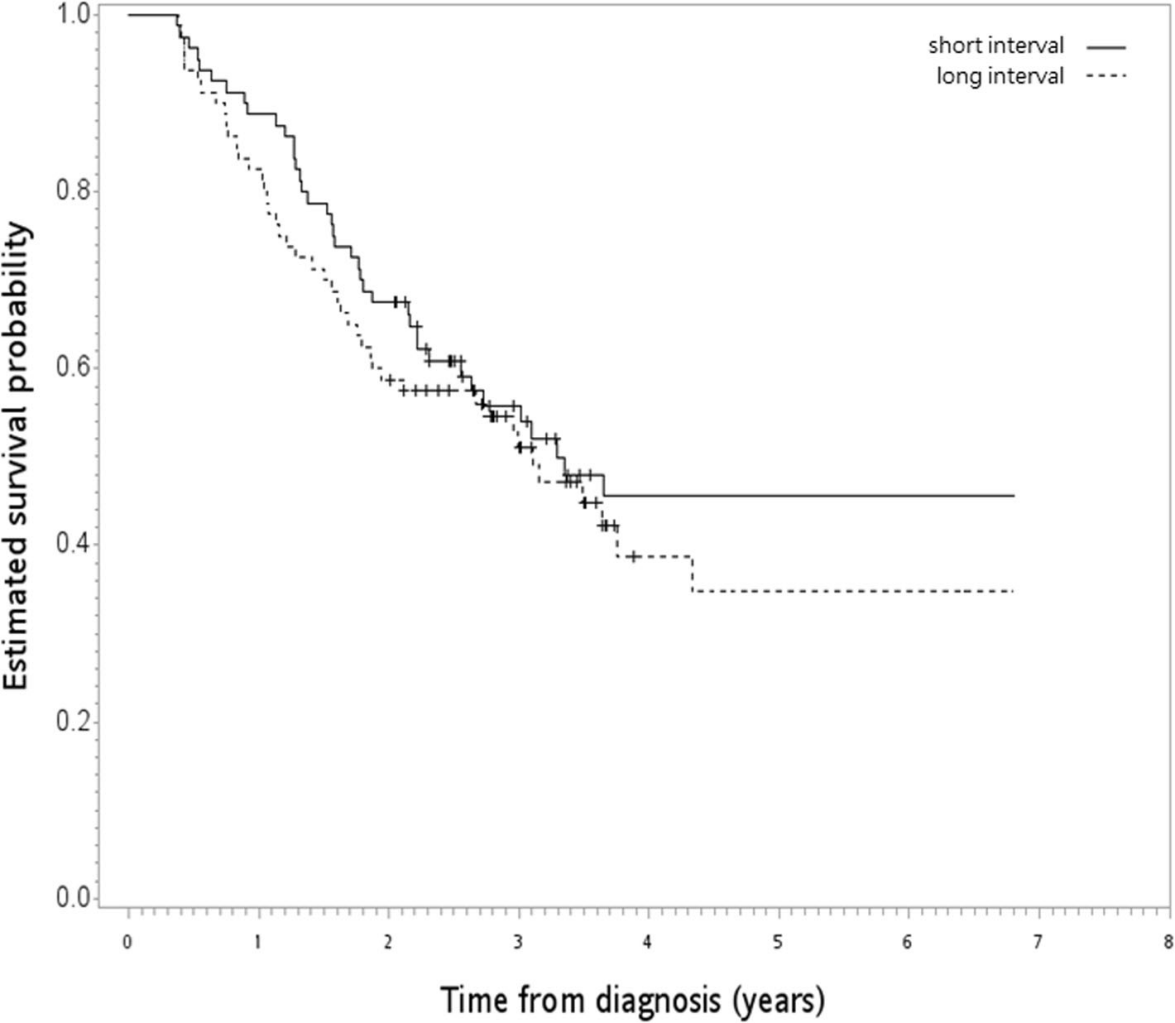
Kaplan–Meier overall survival curve (in years) in the primary analysis

After a median follow-up of 30 months [range 4–81] (median 41 and range 24–81 for the survivors), 83 deaths were recorded (39 and 44 in the short and long interval groups, respectively). The Kaplan–Meier OS curve is shown in Fig. 2. The 1/2/3/4/5-year OS rates [in %] for the short and long interval groups were 89/83, 68/59, 56/51, 45/39, 45/35, respectively. There was no significant difference for HR [1.22; 95% confidence interval (95% CI) 0.78–1.91, *P* = 0.39] when the long interval group was compared to the short interval group. Our result may be due to an unmeasured confounding variable associated with both treatment selection and survival by a risk ratio of 1.56 [*E* value] fold each, but weaker confounding could not do so. The results of the HR for ILRR (HR = 1.44, *P* = 0.29) and IECM (HR = 1.18, *P* = 0.48) were similar. The pCR rates (55% vs 54% for the short vs long interval groups, *P* = 1), 30-day mortality (*P* = 0.06, exact numbers not reported per HWDC policy due to few events), and 90-day mortality (4% vs 9%, *P* = 0.19) were also not significantly different between the two groups.

### Supplementary analysis (SA)

When alternative definitions of the interval between nCCRT and esophagectomy were used, we were still able to construct balanced study populations (Table 2). The results were not significantly different [SA-1: HR for death 1.08, *P* = 0.71; SA-2: HR for death 1.32, *P* = 0.10]. In SA-3, we constructed another balanced study population (Table 3) and found that the results were not significantly different [HR for death 1.22, *P* = 0.35]. There were also no statistically significant differences in the distribution of R0 resection [*P* = 0.07, exact proportion not reported per HWDC policy due to the small number of events]. In SA-4, we constructed additional balanced study population (Table 4) and found that the results were not significantly different [HR for death 1.01, *P* = 0.98].

**Table 2:** Patient characteristics of the study population in the first and second supplementary analyses

		SA-1					SA-2				
		4–8 weeks (*n* = 95)		8–12 weeks (*n* = 95)			4–7 weeks (*n* = 132)		7–12 weeks (*n* = 132)		
		Number or mean (sd)^†^	(%)^†^	Number or mean (sd)^†^	(%)^†^	SDif^†^	Number or mean (sd)^†^	(%)^†^	Number or mean (sd)^†^	(%)^†^	SDif^†^
Age		55.06 (9.29)		54.05 (8.20)		0.115	54.30 (9.13)		54.13 (8.54)		0.019
Gender	Female	7	(7)	6	(6)	0.042	9	(7)	8	(6)	0.031
	Male	88	(93)	89	(94)		123	(93)	124	(94)	
Residency	Non-north	52	(55)	55	(58)	0.064	76	(58)	80	(61)	0.062
	North	43	(45)	40	(42)		56	(42)	52	(39)	
Tumor size	≤ 5 cm	47	(49)	40	(42)	0.148	54	(41)	53	(40)	0.015
	> 5 cm	48	(51)	55	(58)		78	(59)	79	(60)	
Tumor differentiation	Poorly/undifferentiated	21	(22)	21	(22)	0	42	(32)	36	(27)	0.100
	Well/moderately	74	(78)	74	(78)		90	(68)	96	(73)	
RT delivery	Non-IGRT	71	(75)	72	(76)	0.024	103	(78)	104	(79)	0.018
	IGRT	24	(25)	23	(24)		29	(22)	28	(21)	
Use of PET	No	10	(11)	9	(9)	0.035	13	(10)	16	(12)	0.073
	Yes	85	(89)	86	(91)		119	(90)	116	(88)	
Tumor location	Cervical	^‡^	^‡^	^‡^	^‡^	0	^‡^	^‡^	^‡^	^‡^	0
	Non-cervical	^‡^	^‡^	^‡^	^‡^		^‡^	^‡^	^‡^	^‡^	
T-stage	T1-T2	9	(9)	12	(13)	0.101	18	(14)	16	(12)	0.045
	T3-T4	86	(91)	83	(87)		114	(86)	116	(88)	
N-stage	Negative	15	(16)	11	(12)	0.123	16	(12)	14	(11)	0.048
	Positive	80	(84)	84	(88)		116	(88)	118	(89)	
Drinking	No	13	(14)	10	(11)	0.097	16	(12)	15	(11)	0.024
	Yes	82	(86)	85	(89)		116	(88)	117	(89)	
Betel nut chewing	No	45	(47)	39	(41)	0.127	55	(42)	55	(42)	0
	Yes	50	(53)	56	(59)		77	(58)	77	(58)	
Smoking	No	18	(19)	14	(15)	0.113	18	(14)	19	(14)	0.022
	Yes	77	(81)	81	(85)		114	(86)	113	(86)	
BMI		21.85 (3.08)		22.17 (3.21)		0.104	21.83 (3.39)		21.80 (3.19)		0.009
Number of lymph nodes	< 15	19	(20)	24	(25)	0.126	33	(25)	34	(26)	0.017
	≥ 15	76	(80)	71	(75)		99	(75)	98	(74)	
RT dose (Gy)		47.03 (3.97)		47.49 (3.71)		0.120	47.56 (3.68)		47.85 (3.37)		0.080

*BMI* body mass index, *IGRT* image-guided radiotherapy, *nCCRT* neoadjuvant concurrent chemoradiotherapy, *PET* positron emission tomography, *RT* radiotherapy, *sd* standard deviation, *SDif* standardized difference

^†^Rounded

^‡^The exact numbers were not reported because of a Health and Welfare Data Science Center (HWDC) database center policy to avoid numbers in single cells ≤ 2)

**Table 3:** Patient characteristics of the study population in the third supplementary analysis

		Short interval (*n* = 71)		Long interval (*n* = 71)		
		Number or mean (sd)^†^	(%)^†^	Number or mean (sd)^†^	(%)^†^	SDif^†^
Age		54.25 (9.84)		54.01 (8.42)		0.026
Gender	Female	5	(7)	5	(7)	0
	Male	66	(93)	66	(93)	
Residency	Non-north	43	(61)	42	(59)	0.029
	North	28	(39)	29	(41)	
Tumor size	≤ 5 cm	32	(45)	32	(45)	0
	> 5 cm	39	(55)	39	(55)	
Tumor differentiation	Poorly/undifferentiated	17	(24)	18	(25)	0.033
	Well/moderately	54	(76)	53	(75)	
RT delivery	Non-IGRT	55	(77)	57	(80)	0.069
	IGRT	16	(23)	14	(20)	
Use of PET	No	9	(13)	7	(10)	0.089
	Yes	62	(87)	64	(90)	
Tumor location	Cervical	^‡^	^‡^	^‡^	^‡^	0
	Non-cervical	^‡^	^‡^	^‡^	^‡^	
T-stage	T1–T2	9	(13)	9	(13)	0
	T3–T4	62	(87)	62	(87)	
N-stage	Negative	10	(14)	10	(14)	0
	Positive	61	(86)	61	(86)	
Drinking	No	10	(14)	8	(11)	0.085
	Yes	61	(86)	63	(89)	
Betel nut chewing	No	28	(39)	27	(38)	0.029
	Yes	43	(61)	44	(62)	
Smoking	No	15	(21)	13	(18)	0.071
	Yes	56	(79)	58	(82)	
BMI		22.56 (2.73)		22.11 (2.80)		0.164
Number of LNs	< 15	16	(23)	19	(27)	0.098
	≥ 15	55	(77)	52	(73)	
RT dose (Gy)		47.38 (3.93)		47.48 (3.82)		0.025
Patient volume	Low volume	21	(30)	16	(23)	0.161
	High volume	50	(70)	55	(77)	
Positive LN		0.48 (0.91)		0.44 (0.84)		0.048

*BMI* body mass index, *IGRT* image-guided radiotherapy, *LN* lymph node, *nCCRT* neoadjuvant concurrent chemoradiotherapy, *PET* positron emission tomography, *RT* radiotherapy, *sd* standard deviation, *SDif* standardized difference

^†^Rounded

^‡^The exact numbers were not reported because of a Health and Welfare Data Science Center (HWDC) database center policy to avoid numbers in single cells (≤ 2)

**Table 4:** Patient characteristics of the study population in the 4th supplementary analysis

		Short interval (*n* = 63)		Long interval (*n* = 63)		
		Number or mean (sd)^†^	(%)^†^	Number or mean (sd)^†^	(%)^†^	SDif^†^
Age		54.17 (8.22)		54.46 (8.63)		0.034
Gender	Female	^‡^	^‡^	^‡^	^‡^	0
	Male	^‡^	^‡^	^‡^	^‡^	
Residency	Non-north	43	(68)	39	(62)	0.133
	North	20	(32)	24	(38)	
Tumor size	≤ 5 cm	17	(27)	20	(32)	0.105
	> 5 cm	46	(73)	43	(68)	
Tumor differentiation	Poorly/undifferentiated	14	(22)	18	(29)	0.146
	Well/moderately	49	(78)	45	(71)	
RT delivery	Non-IGRT	51	(81)	53	(84)	0.084
	IGRT	12	(19)	10	(16)	
Use of PET	No	9	(14)	8	(13)	0.046
	Yes	54	(86)	55	(87)	
Tumor location	Cervical	^‡^	^‡^	^‡^	^‡^	0
	Non-cervical	^‡^	^‡^	^‡^	^‡^	
T-stage	T1–T2	8	(13)	8	(13)	0
	T3–T4	55	(87)	55	(87)	
N-stage	Negative	6	(10)	6	(10)	0
	Positive	57	(90)	57	(90)	
Drinking	No	8	(13)	8	(13)	0
	Yes	55	(87)	55	(87)	
Betel nut chewing	No	27	(43)	27	(43)	0
	Yes	36	(57)	36	(57)	
Smoking	No	10	(16)	9	(14)	0.044
	Yes	53	(84)	54	(86)	
BMI		21.84 (3.40)		22.07 (3.39)		0.07
Number of LNs	< 15	12	(19)	15	(24)	0.116
	≥ 15	51	(81)	48	(76)	
RT dose (Gy)		48.30 (3.47)		47.59 (3.54)		0.205
Patient volume	Low volume	23	(37)	20	(32)	0.101
	High volume	40	(63)	43	(68)	
Positive LN		0.70 (1.29)		0.75 (1.75)		0.031

*BMI* body mass index, *IGRT* image-guided radiotherapy, *LN* lymph node, *nCCRT* neoadjuvant concurrent chemoradiotherapy, *PET* positron emission tomography, *RT* radiotherapy, *sd* standard deviation, *SDif* standardized difference

^†^Rounded

^‡^The exact numbers were not reported because of a Health and Welfare Data Science Center (HWDC) database center policy to avoid numbers in single cells (≤ 2)

## Discussion

In our analysis of LA-ESqCC treated with nCCRT and esophagectomy in this population-based study from modern Asia, we found that OS was similar for long and short intervals between nCCRT and esophagectomy.

We searched the literature up to May 2019 by using the same strategy as used in the recent systematic review [7] to see if there were other modern studies and found two population-based studies from North America and another two single-institution studies from Asia [25–28]. Azab et al. utilized the American National Cancer Database (NCDB) to identify more than 5000 patients (81% adenocarcinoma) and found that SqCC groups had similar OS across interval lengths [25]. Franko and McAvoy used the same NCDB specifically for SqCC and found that OS was not affected by the interval length [26]. Furukawa et al. identified 134 patients from a Japanese hospital and reported that OS survival rates did not significantly differ between the two groups (≤ 8 or > 8 weeks) [27]. Roh et al. identified 348 Korean patients and found no significant difference in OS between the groups [*P* = 0.101] [28]. Our results were similar to the results of these four studies in that the OS between different interval length groups was similar.

However, there were inherent limitations in our analysis. As in all nonrandomized studies, our results were prone to potential unmeasured confounder(s), although we used PS matching to balance observed covariables. There was a risk of unmeasured confounders (such as surgical techniques or systemic therapy details), so we reported the *E* value, as suggested in the literature [19]. For example, a transthoracic approach has been reported to lead to a trend of favorable long-term outcomes [29] and taxane has been used in modern neoadjuvant trials with excellent results [23]. Besides, the importance of the anastomotic sites or the surgical fields was debated in the literatures [30]. However, these factors were not considered in our study due to the data not being available. Some potential pathological factors like extranodal extension, perineural invasion, or lymphovascular invasion were also not included due to the same data limitation. Therefore, phase III RCTs are needed to clarify the findings from our study and other studies. However, when we searched the clinical trial registry [https://clinicaltrials.gov/] in March 2019 using the keywords “esophagectomy | Interventional Studies | Esophagus Cancer | Phase 3”, we found no relevant studies. Therefore, we believe that our study provides useful information until higher-level data are available.

## Conclusions

In this population-based study from modern Asia, we found that for LA-ESqCC patients treated with nCCRT and esophagectomy, OS was similar for long and short intervals between nCCRT and esophagectomy. Randomized controlled trials are needed to clarify this finding.

## Data Availability

The data that support the findings of this study are available from the Taiwan Cancer Registry, but restrictions apply to the availability of these data, which were used under license for the current study, and so they are not publicly available. Data are, however, available from the authors upon reasonable request and with permission of the Taiwan Cancer Registry.

## Abbreviations

95% CI: 95% confidence interval
BMI: Body mass index
HR: Hazard ratio
HWDC: Health and Welfare Data Science Center
IECM: Incidence of esophageal cancer mortality
IGRT: Image-guided radiotherapy
ILRR: Incidence of local regional recurrence
LA-ESqCC: Locally advanced esophageal SqCC
nCCRT: Neoadjuvant concurrent chemoradiotherapy
NHI: National Health Insurance
OS: Overall survival
PA: Primary analysis
pCR: Pathological complete response
PET: Positron emission tomography
PS: Propensity score
RCT: Randomized controlled trial
RT: Radiotherapy
SA: Supplementary analyses
SDif: Standardized difference
SqCC: Squamous cell carcinoma
TCR: Taiwan Cancer Registry
